# Perturbation effects of the carbon fiber‐PEEK screws on radiotherapy dose distribution

**DOI:** 10.1002/acm2.12046

**Published:** 2017-02-07

**Authors:** Alexander Nevelsky, Egor Borzov, Shahar Daniel, Raquel Bar‐Deroma

**Affiliations:** ^1^ Division of Oncology Rambam Health Care Campus Haifa Israel

**Keywords:** dosimetry, Monte Carlo simulations, spinal implants, spinal radiotherapy

## Abstract

Radiation therapy, in conjunction with surgical implant fixation, is a common combined treatment in cases of bone metastases. However, metal implants generally used in orthopedic implants perturb radiation dose distributions. Carbon‐Fiber Reinforced Polyetheretherketone (CFR‐PEEK) material has been recently introduced for production of intramedullary nails and plates. The purpose of this work was to investigate the perturbation effects of the new CFR‐PEEK screws on radiotherapy dose distributions and to evaluate these effects in comparison with traditional titanium screws. The investigation was performed by means of Monte Carlo (MC) simulations for a 6 MV photon beam. The project consisted of two main stages. First, a comparison of measured and MC calculated doses was performed to verify the validity of the MC simulation results for different materials. For this purpose, stainless steel, titanium, and CFR‐PEEK plates of various thicknesses were used for attenuation and backscatter measurements in a solid water phantom. For the same setup, MC dose calculations were performed. Next, MC dose calculations for titanium, CFR‐PEEK screws, and CFR‐PEEK screws with ultrathin titanium coating were performed. For the plates, the results of our MC calculations for all materials were found to be in good agreement with the measurements. This indicates that the MC model can be used for calculation of dose perturbation effects caused by the screws. For the CFR‐PEEK screws, the maximum dose perturbation was less than 5%, compared to more than 30% perturbation for the titanium screws. Ultrathin titanium coating had a negligible effect on the dose distribution. CFR‐PEEK implants have good prospects for use in radiotherapy because of minimal dose alteration and the potential for more accurate treatment planning. This could favorably influence treatment efficiency and decrease possible over‐ and underdose of adjacent tissues. The use of such implants has potential clinical advantages in the treatment of bone metastases.

## 1 Introduction

The optimal treatment of spinal and para‐spinal tumors necessitates a multidisciplinary approach, including surgery and radiation (adjuvant) therapy. Surgery plays a primary management role and permanent metallic hardware is often required.^1^ Spinal fixation, using pedicle screws and implant rods, has recently been used for rigid connections in the vertebrae of the spine.^2^ Radiotherapy is administered postoperatively to improve local control, especially in the setting of close margins or microscopically positive margins.^3^

Unfortunately, delivery of adjuvant radiation therapy can be compromised due to the presence of the postoperative hardware. Traditional orthopedic implant materials are usually stainless steel or titanium and their use poses two major obstacles for accurate radiotherapy planning and delivery.

First, the CT images of patients with metallic implants contain artifacts. Since electron densities of various tissues in the body are calculated from the Hounsfield Unit (HU) values of computed tomography (CT) images and those are contaminated by the image artifacts, dose calculations performed in treatment planning systems (TPS) may contain errors that can be beyond a clinically acceptable range.^4^

Second, metal implants distort dose distributions from therapeutic megavoltage beams. A 5% to 10% dose reduction to tissues in regions behind stabilization rods has been reported, due to the attenuation effect.^5^, ^6^, ^7^ As a result, some patients who might benefit from radiotherapy may be treated with lower doses unlikely to provide long‐term local control. On the other hand, enhanced scattering from the high‐Z materials^8^ in combination with high doses of radiation prescription may lead to the development of myelopathy, the most feared complication of radiotherapy.

Approaches to reducing photon dose calculation errors near metal implants were described in a recent publication^9^ and are out of the scope of this paper. However, it should be noted that correct dose calculation in the vicinity of metal implants is still problematic, even with the use of modern TPS and new image artifacts reduction methods.

Unlike metals, carbonaceous materials have low atomic numbers, good biocompatibility, chemical stability, good mechanical properties, and modulus of elasticity similar to human bones. Carbon‐Fiber Reinforced Polyetheretherketone (CFR‐PEEK) material has been recently introduced for production of intramedullary nails and plates and now is broadly accepted as a radiolucent alternative to metallic biomaterials in the spine community.^10^, ^11^, ^12^ Using implants made of CFR‐PEEK materials can eliminate the problem of imaging artifacts, as well as the problem of dose perturbation in post implantation radiotherapy.

Recently, a novel pedicle screws and rods system comprised of continuous CFR‐PEEK was developed (CarboFix Orthopedics Ltd., Herzeliya, Israel). The purpose of this work was to investigate the perturbation effects of the new CFR‐PEEK screws on radiotherapy dose distributions and to evaluate these effects in comparison with traditional titanium screws. The investigation was performed by means of Monte Carlo (MC) simulations for a 6 MV photon beam.

## 2 Methods/Materials

### 2.A General description

The project consisted of two main stages. First, a comparison of measured and MC calculated doses was performed to verify the validity of the MC simulation results for different materials. For this purpose, stainless steel, titanium, and CFR‐PEEK plates of various thicknesses were used for attenuation and backscatter measurements in a solid water phantom. For the same setup, MC dose calculations were performed. Next, MC dose calculations for titanium and CFR‐PEEK screws were performed. The screw axis was either parallel or perpendicular to the beam axis. Dose perturbation was assessed for both situations.

### 2.B Measurements

Stainless steel, titanium, and carbon fiber plates of 1, 3, and 6 mm thicknesses placed in a RMI‐457 solid water phantom (GAMMEX RMI, Middleton, WI, USA) were used for attenuation and backscatter measurements at SSD = 100 cm and a 10 × 10 cm^2^ field. Solid water phantom dimensions were 30 × 30 × 20 cm^3^. The plates were placed at a depth of 6 cm, as this is a representative depth of spinal cord for posterior irradiation. The attenuation was measured with a plane‐parallel Roos PTW ion chamber (PTW, Freiburg, Germany) at distances of 1, 3, and 6 mm from the exit plate surface. For the backscatter measurements, the Roos ion chamber was used in the upside down position. One hundred MUs were delivered for each measurement. As there is only minor dependence of backscatter effect on the plate thickness observed from MC calculations, the measurements were performed only for the plates of 3 mm thickness, at distances of 1, 3, and 6 mm from the entrance plate surface.

### 2.C MC simulations

All MC simulations in this study were performed with the EGSnrc code^13^ for coupled electron and photon transport. This code has been extensively validated in the past and was selected as best suited for our study. Unlike commercial TPS, EGSnrc code is capable of accurate dose calculations near metal objects as it fully models all physical interactions of ionizing particles with the medium. BEAMnrc^14^ is an EGSnrc‐based package that allows for the simulation of radiotherapy treatment units using predefined component modules. BEAMnrc code was used to simulate the 6 MV photon beams of the Elekta Precise linear accelerator (Elekta AB, Stockholm, Sweden). The modeled geometry for the Elekta Precise linac was entered into the BEAMnrc code using full details of the linac head provided by the manufacturer. Only parts exposed to the radiation beam were modeled. To guarantee good statistical accuracy of dose simulations, 10^8^ primary electrons were transported from the exit window. The DOSXYZnrc^15^ code was used for the dose calculation in a phantom.

The cut‐off energies for photon transport (P_cut)_ and for electron transport (E_cut_) were set to 0.01 MeV and 0.7 MeV, respectively, in both accelerator simulation and phantom dose calculation. For all simulations, the boundary crossing algorithm was EXACT and the electron step algorithm was PRESTA‐II. The user adjustable values for other parameters were set at their default values.

Spin effect (which turns off/on spin effects for electron elastic scattering) was set to ON, as it is necessary for accurate backscattering calculations.^15^

### 2.D Screws

**Figure 1:**
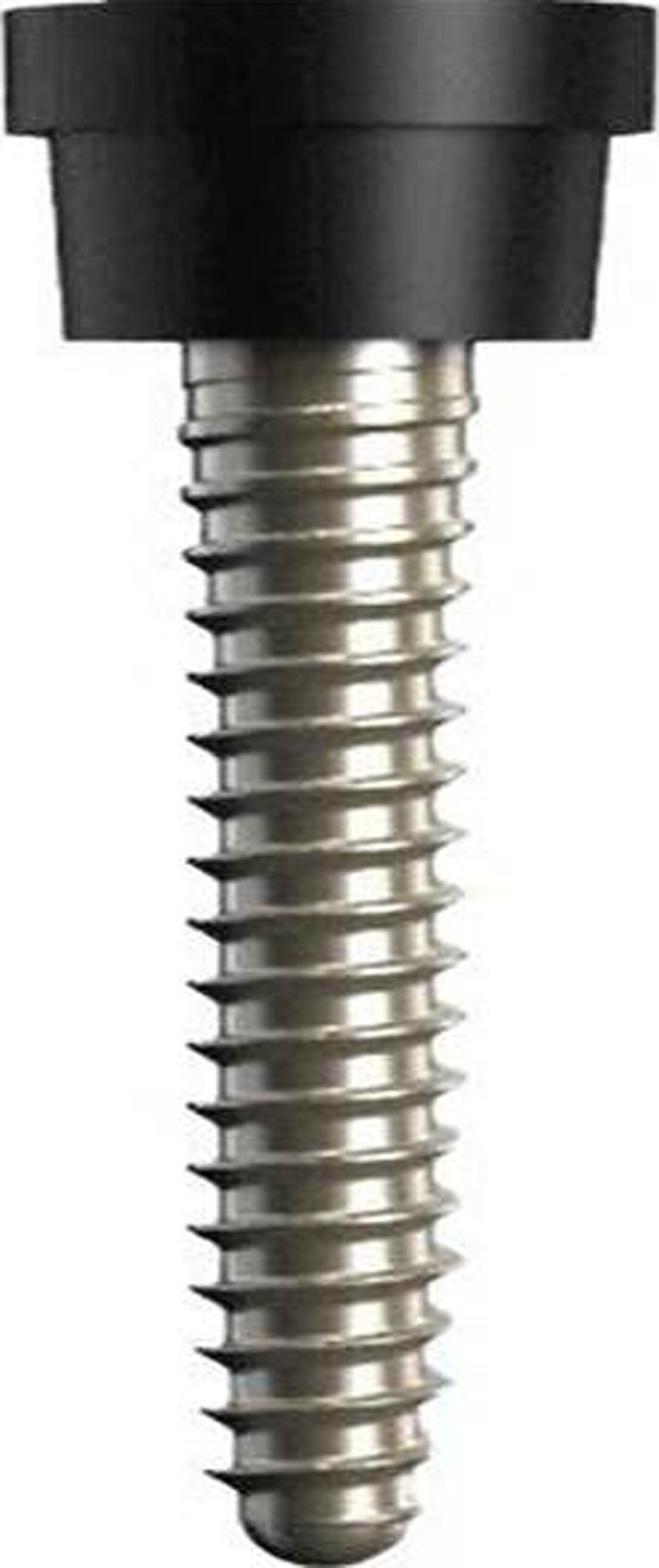
CFR‐PEEK pedicle screw with thin titanium coating.

The screws investigated in this work are shown in Fig. 1. The screws were modeled as 0.6 cm × 0.6 cm × 6 cm parallelepiped. Three screw compositions were studied in this work. At the first stage, homogeneous screws made of titanium and CFR‐PEEK were considered. Then, CFR‐PEEK screws with ultrathin (thickness of less than 0.01 cm) titanium coating were modeled. Titanium coating is used for improved and direct osseointegration.^16^ In addition, this titanium shell improves screw visibility under fluoroscopy.

### 2.E Assessment of dose perturbation due to the screw presence

MC dose calculations in a 10 cm × 10 cm × 12 cm water phantom with and without the presence of the pedicle screws were performed. The phantom size was selected so that the screw depth would be representative of the spinal implants. To provide good scattering conditions, water slabs of 5 cm surrounding the phantom were added in DOSXYZnrc. The screw was placed in the center of the phantom. Beam field size was 10 cm × 10 cm, with the SSD kept constant and equal to 100 cm. The beam entrance direction was changed so that the screw axis was either parallel or perpendicular to the beam axis, as these orientations would introduce minimal and maximal dose perturbations.

**Figure 2:**
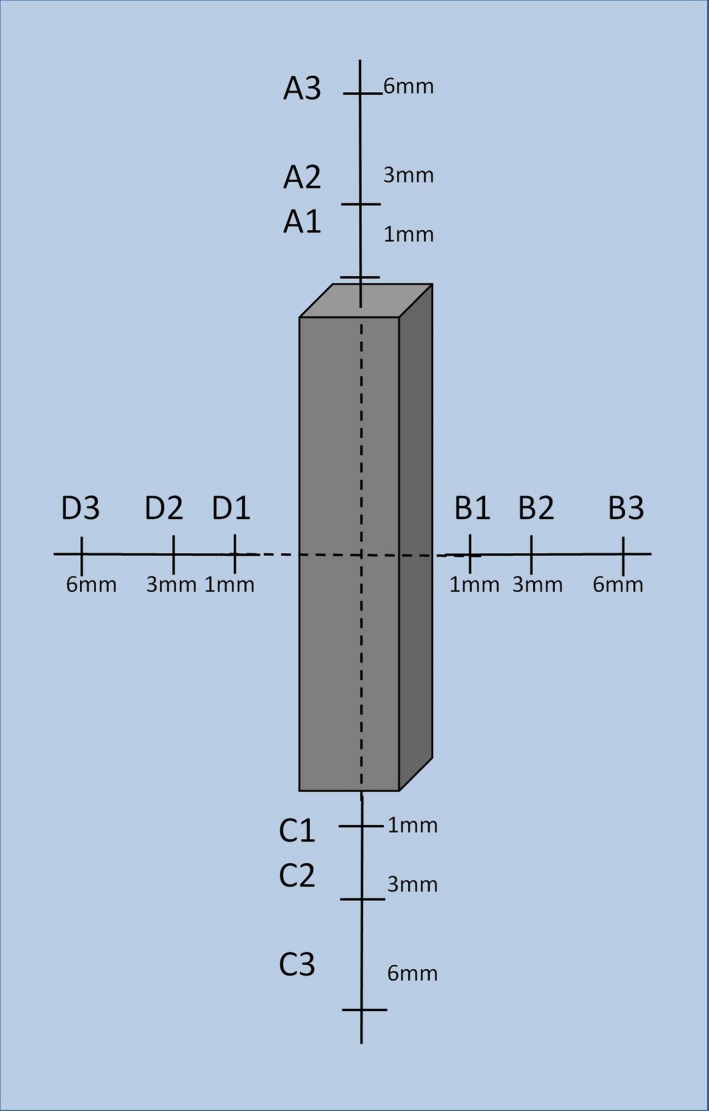
Definition of points around the screw model where the dose difference is reported.

First, MC simulations of dose distribution were performed for a water phantom without a screw presence. In this simulation, the maximum dose was defined and the doses were normalized to this value, yielding percentage dose distributions. Then MC simulations were performed in a water phantom with a screw, for every screw composition, and the doses were also normalized to the maximum dose in the simulation without the screw. Finally, maps of percentage dose difference for distribution in the water phantom with and without a screw were built for every screw composition, by subtraction of percentage doses in each voxel between the corresponding dose distributions. This approach allows excluding effects of dose attenuation in water and evaluating dose perturbation due to the screw presence only. For the quantitative analysis of the dose perturbation with introduction of different screws relative to a homogeneous water phantom, the percentage dose differences at a number of points around the screw were calculated. Figure 2 presents a description of the points.

## 3 Results

### 3.A MC simulations and measurements for the plates

**Figure 3:**
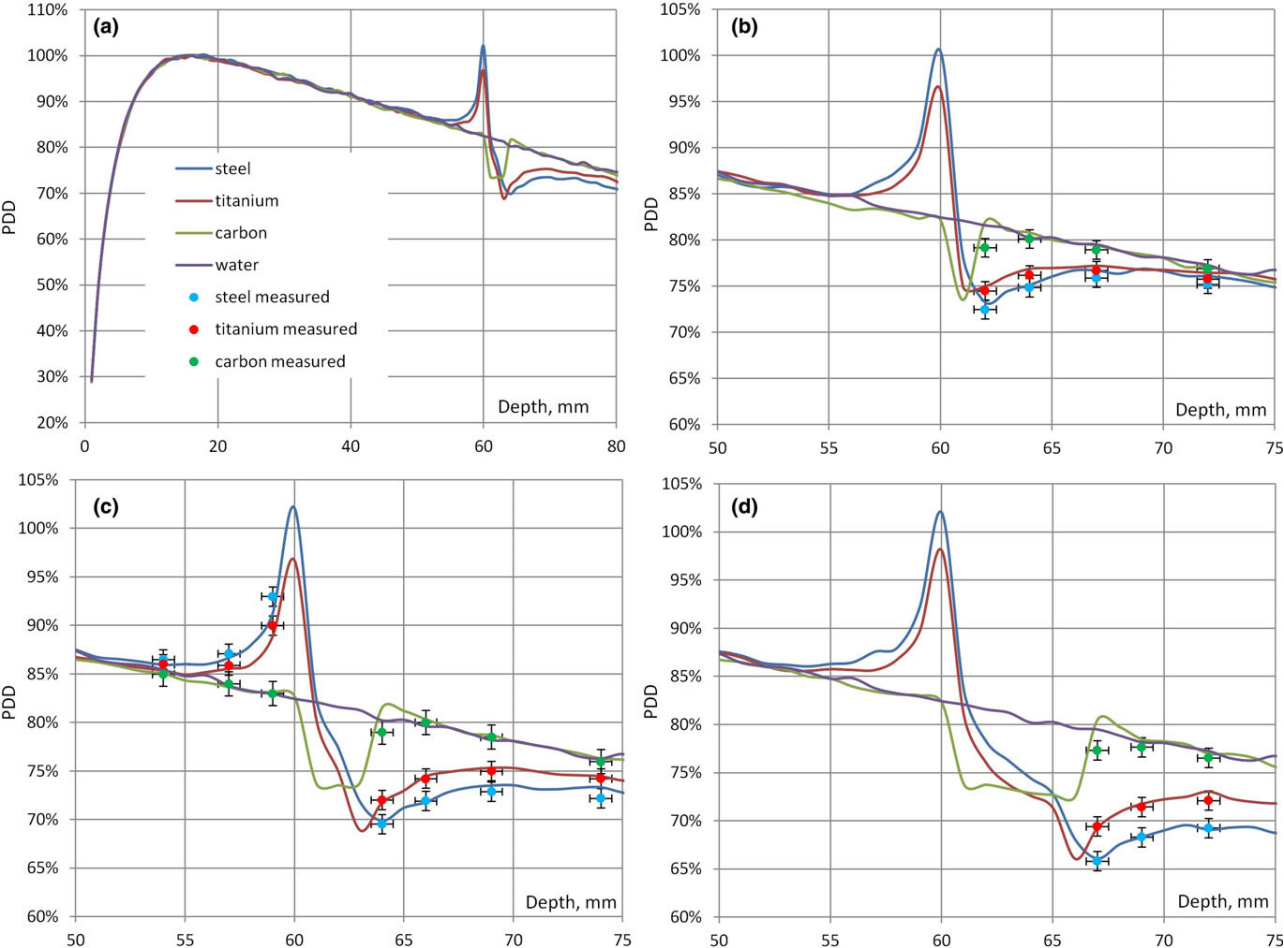
(a) PDD curves in water with and without the presence of 3 mm plates of steel, titanium, CFR‐PEEK shown on the larger depth scale; (b) PDD curves and attenuation measurements for 1 mm plates; (c) PDD curves, attenuation and backscatter measurements for 3 mm plates; (d) PDD curves and attenuation measurements for 6 mm plates.

MC calculated percent depth dose curves in water for 1 mm, 3 mm, and 6 mm plates of steel, titanium and CFR‐PPEK are shown in Figs. 3(b), 3 (c), and 3 (d) correspondingly). The vertical and the horizontal error bars correspond to ± 1% variation in the dose and ± 0.5 mm variation in the distance, respectively. The error bars were added to the measured data points to allow an easy visual comparison between the results.

The dose at the entrance surface of the plates was increased (the effect of backscatter) by 22% for stainless steel, 18% for titanium, and less than 1% for CFR‐PEEK. There was only minor dependence (less than 1%) on the thickness of the plate. For the same plates, the dose at the exit surface was reduced by 10, 13, and 17% for steel and by 8, 10, and 13% for titanium for the thickness of 1, 3, and 6 mm, correspondingly. For the CFR‐PEEK plates, the dose was increased by less than 2%.

The results of attenuation measurements are shown in Fig. 3, together with MC calculated percent depth dose curves in water for 1 mm, 3 mm, and 6 mm plates of steel, titanium, and CFR‐PEEK [Figs. 3 (b), 3 (c), and 3 (d), correspondingly]. For the 3 mm plates, the results of backscatter measurements are also shown [Fig. 3 (c)]. It can be seen that all measurements were within 1%/1 mm agreement with the MC calculations.

### 3.B MC simulations for the screws

**Figure 4:**
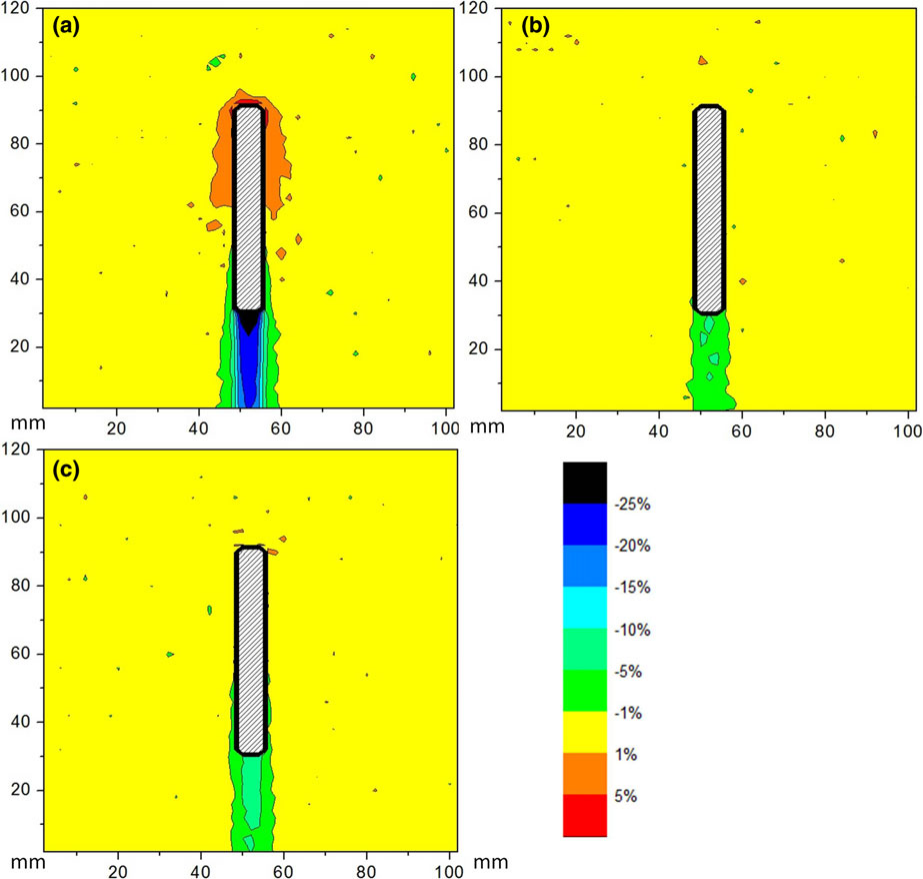
Map of the dose difference for the screws made of (a) Titanium, (b) CFR‐PEEK, (c) CFR‐PEEK‐TI; beam comes from above.

**Figure 5:**
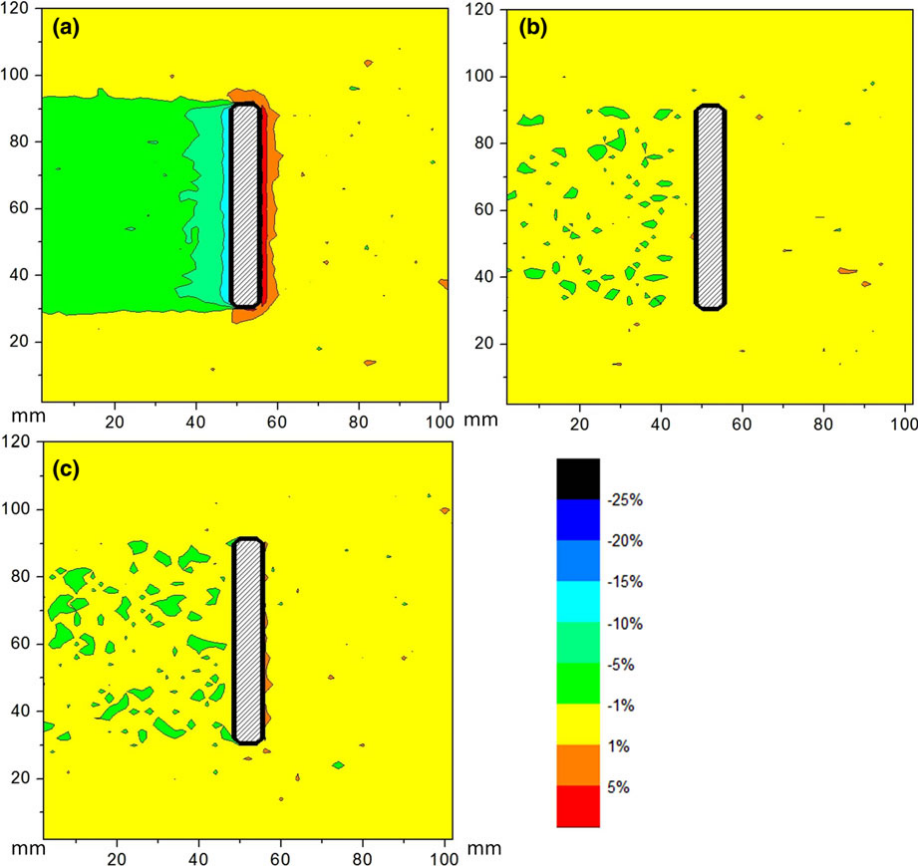
Map of the dose difference for the screws made of (a) Titanium, (b) CFR‐PEEK, (c) CFR‐PEEK‐TI; beam comes from the right side.

Dose difference maps for screws made of titanium (TI), CFR‐PEEK, and CFR‐PEEK with titanium coating (CFR‐PEEK‐TI) are shown in Figs. 4 and 5, for the beam coming along (from the top) and across (from the right) the screws, respectively. Dose differences at the points defined in Fig. 2 are summarized in Table 1. Zero value means that observed dose difference was < 0.5%.

**Table 1:**
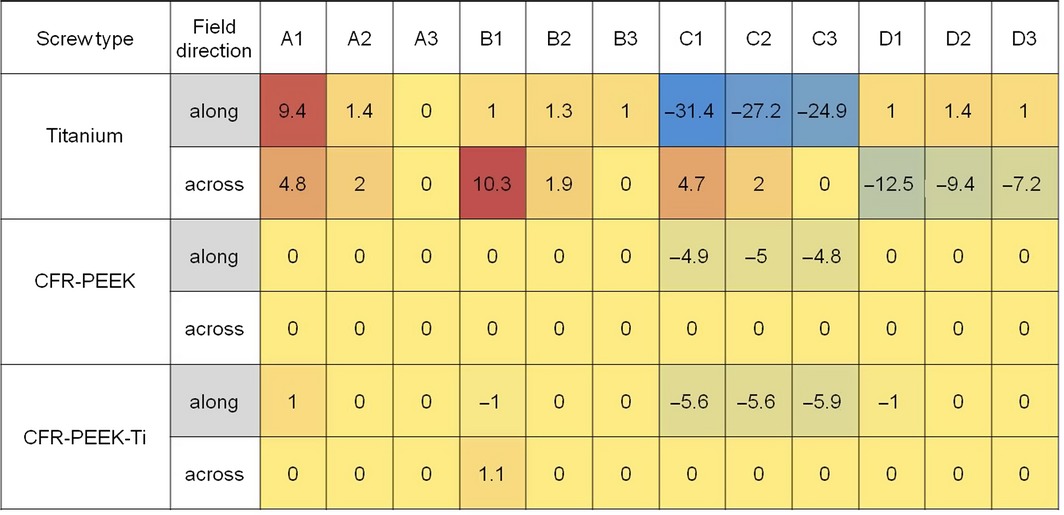
Dose differences in percents at the points defined in Fig. 2.

## 4 Discussion

In this study, we employed MC simulations to investigate dose perturbation effects in 6 MV photon beam caused by pedicle screws of different compositions. To validate the MC model, we compared measurements and MC calculations for plates of three different materials and three different thicknesses. The results of our MC calculations for the stainless steel, titanium, and CFR‐PEEK plates were in good agreement with the measurements for all materials. This indicates that the MC model can be used for the calculation of dose perturbation effects caused by the screws. For the 3 mm plates, MC calculated dose at the entrance and at the exit surface were in close agreement with the findings of Ni et al.^17^ Dose increase due to the backscatter effect depends mainly on the material of the plate, while dose reduction due to attenuation depends both on the material and on the thickness of the plate.

Maximum overdose due to backscatter was 10% for the Ti screws and effectively zero for the CFR‐PEEK screws (both with and without titanium coating). Maximum underdose due to attenuation was about 30% for the Ti screws and about 5% for the CFR‐PEEK screws. As expected, the largest perturbation occurred when the beam direction was along to the screw axis.

**Figure 6:**
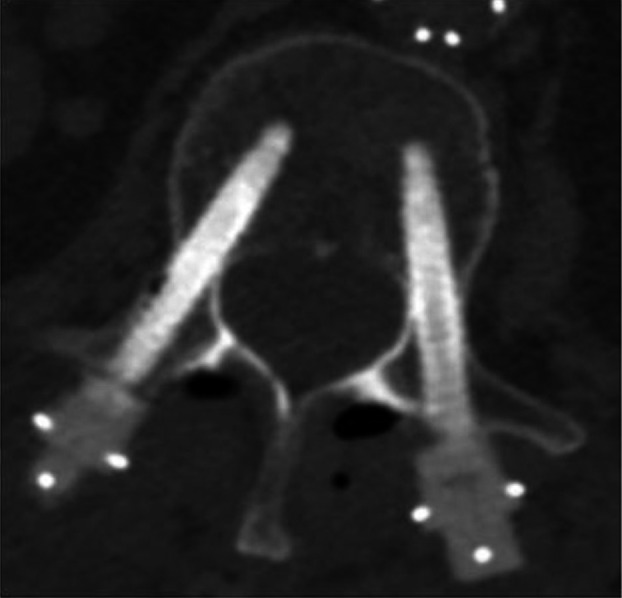
CT image of pedicle screws with titanium coating inserted into vertebra.

It can be seen that the dose overdose due to backscatter decreases quickly with distance and becomes negligible at a few millimeters from the screw. However, there might be situations where screws are placed in very close vicinity of the spinal cord, as illustrated in Fig. 6. It can also be seen here that the ultrathin titanium shell allows acquisition of CT images with good screw visualization and minimum appearance of image arifacts.

Titanium screws introduced large distortion in the radiation dose distribution. CFR‐PEEK screws caused minimal alteration of dose distribution. Ultrathin titanium coating had a negligible effect on the dose distribution.

Only two scenarios were considered in this work, when screws are either parallel or perpendicular to the beam directions. These scenarios correspond to the maximum and minimum perturbations introduced by the screws; when screws are oriented at other angles to the beam, dose perturbation will be within the range of the minimum and maximum values given in our results for perpendicular and parallel screw orientations.

In our work, we concentrated on the dose perturbation due to the screw presence. Image artifacts which can influence clinical dosimetry were not considered here and should be investigated separately.

Unlike the recent publication of Ni et al.,^17^ our work concentrated on the comparison of perturbation effects of pedicle screws with different compositions, as these effects can be of special importance due to proximity to the spinal cord. This comparison was performed for 6 MV photon beam only, because dose perturbation effects of metal implants are even greater for higher photon energies used in radiotherapy^18^ and, therefore, the advantages of CFR‐PEEK screws over metal screws would be more pronounced.

## 5 Conclusion

CFR‐PEEK implants have good prospect for use in radiotherapy because of minimal dose alteration and the potential for more accurate treatment planning. This could favorably influence treatment efficiency and decrease possible over‐ and underdose of adjacent tissue. The use of such implants has potential clinical advantages in the treatment of neoplastic bone disease.
